# Duchenne muscular dystrophy treatment with lentiviral vector containing mini‐dystrophin gene in vivo

**DOI:** 10.1002/mco2.423

**Published:** 2024-01-06

**Authors:** Xiaoyu Wang, Yanghui Zhu, Taiqing Liu, Lingyan Zhou, Yunhai Fu, Jinhua Zhao, Yinqi Li, Yeteng Zheng, Xiaodong Yang, Xiangjie Di, Yang Yang, Zhiyao He

**Affiliations:** ^1^ Department of Pharmacy Cancer Center and State Key Laboratory of Biotherapy West China Hospital Sichuan University Chengdu Sichuan China; ^2^ Clinical Trial Center/NMPA Key Laboratory for Clinical Research and Evaluation of Innovative Drug West China Hospital Sichuan University Chengdu Sichuan China; ^3^ Key Laboratory of Drug‐Targeting and Drug Delivery System of the Education Ministry Sichuan Engineering Laboratory for Plant‐Sourced Drug and Sichuan Research Center for Drug Precision Industrial Technology, West China School of Pharmacy Sichuan University Chengdu Sichuan China

**Keywords:** Duchenne muscular dystrophy, dystrophin, gene delivery, gene therapy, lentiviral vector

## Abstract

Duchenne muscular dystrophy (DMD) is an incurable X‐linked recessive genetic disease caused by mutations in the dystrophin gene. Many researchers aim to restore truncated dystrophin via viral vectors. However, the low packaging capacity and immunogenicity of vectors have hampered their clinical application. Herein, we constructed four lentiviral vectors with truncated and sequence‐optimized dystrophin genes driven by muscle‐specific promoters. The four lentiviral vectors stably expressed mini‐dystrophin in C2C12 muscle cells in vitro. To estimate the treatment effect in vivo, we transferred the lentiviral vectors into neonatal C57BL/10ScSn‐*Dmd^mdx^* mice through local injection. The levels of modified dystrophin expression increased, and their distribution was also restored in treated mice. At the same time, they exhibited the restoration of pull force and a decrease in the number of mononuclear cells. The remissions lasted 3–6 months in vivo. Moreover, no integration sites of vectors were distributed into the oncogenes. In summary, this study preliminarily demonstrated the feasibility and safety of lentiviral vectors with mini‐dystrophin for DMD gene therapy and provided a new strategy to restore truncated dystrophin.

## Graphical abstract

We constructed four lentiviral vectors that could restore mini‐dystrophin expression, dystrophin distribution, and pull force in vivo. Also they reduced the number of mononuclear cells in the treated mice. This study preliminarily demonstrated the feasibility and safety of lentiviral vectors with mini‐dystrophin for DMD gene therapy.

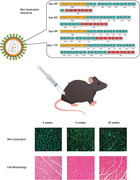

## 1 INTRODUCTION

Duchenne muscular dystrophy (DMD) is an X‐linked recessive genetic disease that affects approximately one in every 5000−6000 male newborns.^1^, ^2^ Most of the patients manifest difficulties with climbing, a waddling gait, frequent falls at 2−3 years old, and a life‐limiting muscle‐wasting condition.^3^, ^4^ On account of the advancements in management, the patients’ median overall survival has been extended.^5^, ^6^

DMD is caused by mutations in the gene encoding dystrophin. In muscles, dystrophin forms a complex with cytoskeletal actin, sarcolemmal‐associated glycoproteins, and neuronal nitric oxide synthase.^7^, ^8^ The sarcolemmal‐associated glycoprotein complex in turn links to the surrounding extracellular matrix to increase muscle strength and prevent muscle fiber injury. Mutations in the DMD gene can lead to different phenotypes.^9^ Frameshift mutations or nonsense mutations that result in a premature stop codon always lead to the expression of nonfunctional or unstable dystrophin. These present as DMD. The point mutations or in‐frame deletions/duplications that result in slight property changes or dysfunction present as a milder clinical phenotype, which is called Becker muscular dystrophy.^10^

Currently, a range of treatment options include glucocorticoids, small‐molecule medication, and oligomeric antisense nucleotides.^11^ Glucocorticoids, such as prednisone (Deltasone) and deflazacort (Emflaza), could reduce inflammation and increase total muscle mass and strength. Consequently, they improve cardiopulmonary function, reduce the need for spinal surgery, and delay the development of cardiac disease. Small‐molecule medication ataluren (Translarna) is administered to skip the terminators produced by mutations and express functional dystrophin isoforms, thus alleviating disease progression. Oligomeric antisense nucleotides, such as golodirsen (Vyondys 53),^12^, ^13^ casimersen (Amondys 45),^14^, ^15^ and eteplirsen (Exondys 51),^16^ specifically recognize the pre‐mRNA and bind with it.^17^ As a result, they promote the expression of functional dystrophin and play a therapeutic role. However, it is currently understood that none of the three types of medications have been found to provide a complete cure for DMD. Furthermore, none of them was applied to all phenotypes. They were also associated with several side effects and repeated administrations. In contrast, gene replacement therapy had the advantages of long‐term effects and radical cures.^1^, ^18^ For example, Mendell et al.^19^ and Potter et al.^20^ demonstrated that the adeno‐associated virus (AAV) vector (rAAVrh74.MHCK7.micro‐dystrophin) could be used to treat DMD. Although the therapeutic benefit of gene replacement has been demonstrated in recent years, little attention has been paid to the virus vectors. The AAV vector is a commonly used vector in DMD gene therapy,^21^ yet immunogenicities, preexisting serum antibodies,^22^ and low packaging capacity still hamper their clinical applications.^23^, ^24^ Apart from a wide application in the hemoglobin disease,^25^, ^26^ leukemia,^27^, ^28^ and primary immunodeficiency syndrome,^29^, ^30^ the lentiviral vector (LV) also provides larger packaging capacity and lower immunogenicity.^31^

In this work, we used LVs that contained modified human dystrophin genes driven by muscle‐specific promoters in gene replacement therapy. First, we analyzed their effects on C2C12 muscle cells. Then, we injected the LVs into the muscle tissues of mdx (C57BL/10ScSn‐*Dmd^mdx^*/J) mice. We observed the mini‐dystrophin expression level and muscle strength to evaluate their therapeutic effects in vivo. Finally, we observed the vector integration sites via deoxyribonucleic acid sequencing technologies.

## 2 RESULTS

### 2.1 Designed and constructed the mini‐dystrophin coding sequences

**FIGURE 1:**
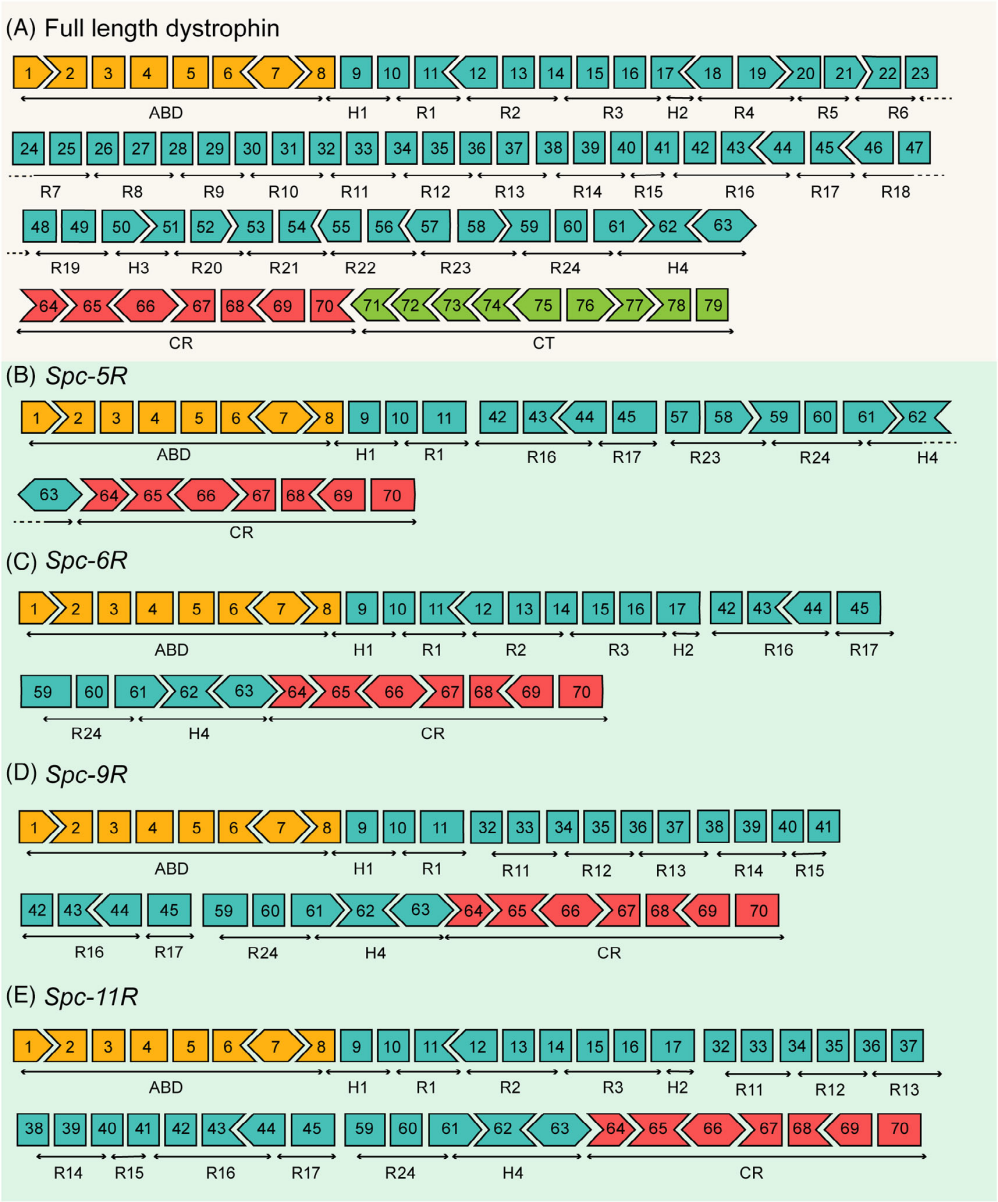
Schematic illustration of full‐length dystrophin and mini‐dystrophin coding sequences. (A) The full‐length protein included four major structural domains: an F‐acting‐binding domain (ABD; encoded by exons 1−8), a central rod domain (encoded by exons 8−64), a cysteine‐rich domain (CR; encoded by exons 64−70), and a distal C‐terminal domain (CT; encoded by exons 71−79). The central rod domain could be divided into 24 spectrin‐like repeats (R1–R24) and four hinges (H1–H4). *Spc‐5R*, *Spc‐6R*, *Spc‐9R*, and *Spc‐11R* were modified from full‐length dystrophin. (B) The *Spc‐5R* sequence lacked the R2–R15, R18–R22, H2–H3, and C‐terminal domains. (C) The *Spc‐6R* sequence lacked the R4–R15, R18–R23, H3, and C‐terminal domains. (D) The *Spc‐9R* sequence lacked the R2–R10, R18–R23, H2–H3, and C‐terminal domains. (E) The *Spc‐11R* sequence lacked the R4–R10, R18–R23, H3, and C‐terminal domains. All four mini‐dystrophin sequences underwent codon optimization and were driven by the *Spc‐5‐12* promoter.

Previous studies demonstrated that a five‐repeat micro‐dystrophin gene could alleviate the DMD progression and restore muscle function in the severe DBA/2J‐mdx model.^32^ In this study, the four modified human dystrophin gene sequences contained more genetic information for better therapeutic effects. The binding of the R1–R3 domains with the sarcolemma was added to modified sequences. The muscle‐specific promoter *Spc‐5‐12* was used to drive the expression of modified sequences.^33^, ^34^ Figure 1 depicts the diagrams of full‐length (Figure 1A) and modified dystrophin (Figure 1B–E).

This study designed coding sequences of four mini‐dystrophin proteins: *Spc‐5R*, *Spc‐6R*, *Spc‐9R*, and *Spc‐11R*. The translation product of the *Spc‐5R* sequence (Figure 1B) was the same as that of the five‐repeat micro‐dystrophin sequence constructed by Hakim et al.,^32^ whose micro‐dystrophin has achieved disease rescue in severe DMD model mice. As can be observed, the *Spc‐6R* sequence (Figure 1C) contains the R1–R3 sequences, which are directed to bind with the sarcolemma.^35^ From this, the translation product of *Spc‐6R* might exhibit better sarcolemma localization properties, which could transform into better therapeutic effects. The *Spc‐9R* sequence (Figure 1D) contained the R11–R15 domains, which directly interacted with F‐actin and sarcolemma.^35^ The *Spc‐11R* sequence (Figure 1E) contains both the R1–R3 and R11–R15 domains, which might be better at preventing mechanical force damage. Codon optimization was performed in all of the nucleotide sequences for higher expression levels in mammals. For the more complete genetic information, the modified dystrophin sequence might be slightly larger. The larger packaging ability of a gene vector such as LVs could assist in this issue. Therefore, these sequences were constructed into lentiviral plasmids. For the long‐term effect and less frequent dosing, LVs were appropriately used to deliver the modified genetic sequences into muscle cells. In summary, the modified dystrophin sequences and LVs might achieve better therapeutic effects in the gene therapy of DMD.

### 2.2 LV‐mediated modified dystrophin expression in C2C12 myoblast cells

**FIGURE 2:**
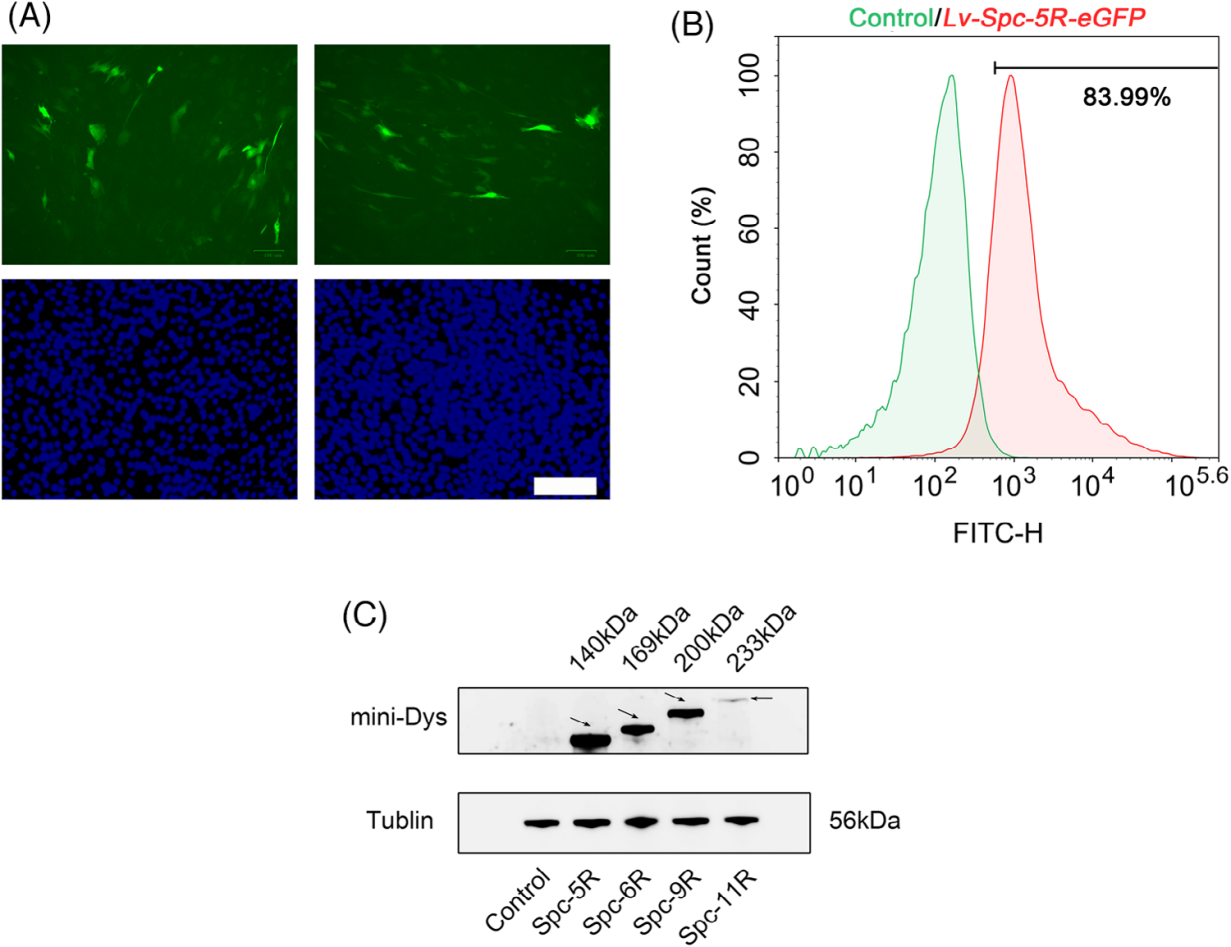
C2C12 myoblast cells transduced with lentiviral vectors achieved modified dystrophin expression in vitro. (A) Fluorescent expression obtained from C2C12 cells after lentiviral vector treatment. The multiplicity of infection was 100 in the left graph and 50 in the right. Green positive cells indicate successful transfection by vectors. Nuclei were stained with DAPI dihydrochloride (blue). Scale bar = 200 μm. (B) The transfection efficiency of *Lv‐Spc‐5R‐eGFP* was analyzed by flow cytometry. Of the treated cells, 83.99% (*Lv‐Spc‐5R‐eGFP*, red) were GFP‐positive compared with the control group (green). (C) At 72 h after treatment with *Lv‐Spc‐5R*, *Lv‐Spc‐6R*, *Lv‐Spc‐9R*, and *Lv‐Spc‐11R*, the expression of mini‐dystrophin was detected by western blot. The target band is indicated by the black arrowheads. The dystrophin levels of the *Lv‐Spc‐5R*, *Lv‐Spc‐6R*, and *Lv‐Spc‐9R* groups appreciably increased. Control: solvent‐treated group. Spc‐5R, Spc‐6R, Spc‐9R, and Spc‐11R: *Lv‐Spc‐5R*‐, *Lv‐Spc‐6R*‐, *Lv‐Spc‐9R*‐, and *Lv‐Spc‐11R*‐treated groups, respectively. eGFP, enhanced green fluorescence protein.

To explore whether the LVs could transfer the modified genetic sequences into muscle cells, the levels of modified dystrophin in C2C12 muscle cells transduced by LVs were examined. The results were divided into three parts, as follows. The level of green fluorescence protein (GFP) was observed by fluorescence microscopy in C2C12 myoblast cells (Figure 2A), which were transduced by LVs (*Lv‐Spc‐5R‐eGFP*). The transduction efficiency was analyzed by flow cytometry (Figure 2B). The modified dystrophin expression level of transduced C2C12 cells was detected by western blot (Figure 2C). As illustrated in Figure 2A–C, green fluorescence is presented in transduced cells in vitro. According to Figure 2B, the transduction efficiency was acceptable at 83.99%. Figure 2C displays the western blot results. It could be observed that *Lv‐Spc‐5R/6R/9R* achieved distinct modified dystrophin expression. The band for full‐length *Spc‐11R* (233 kDa) was faint. This might be associated with larger coding sequence. In conclusion, LVs could be used for gene therapy for DMD.

### 2.3 Modified dystrophin expression following in vivo LV treatment

**FIGURE 3:**
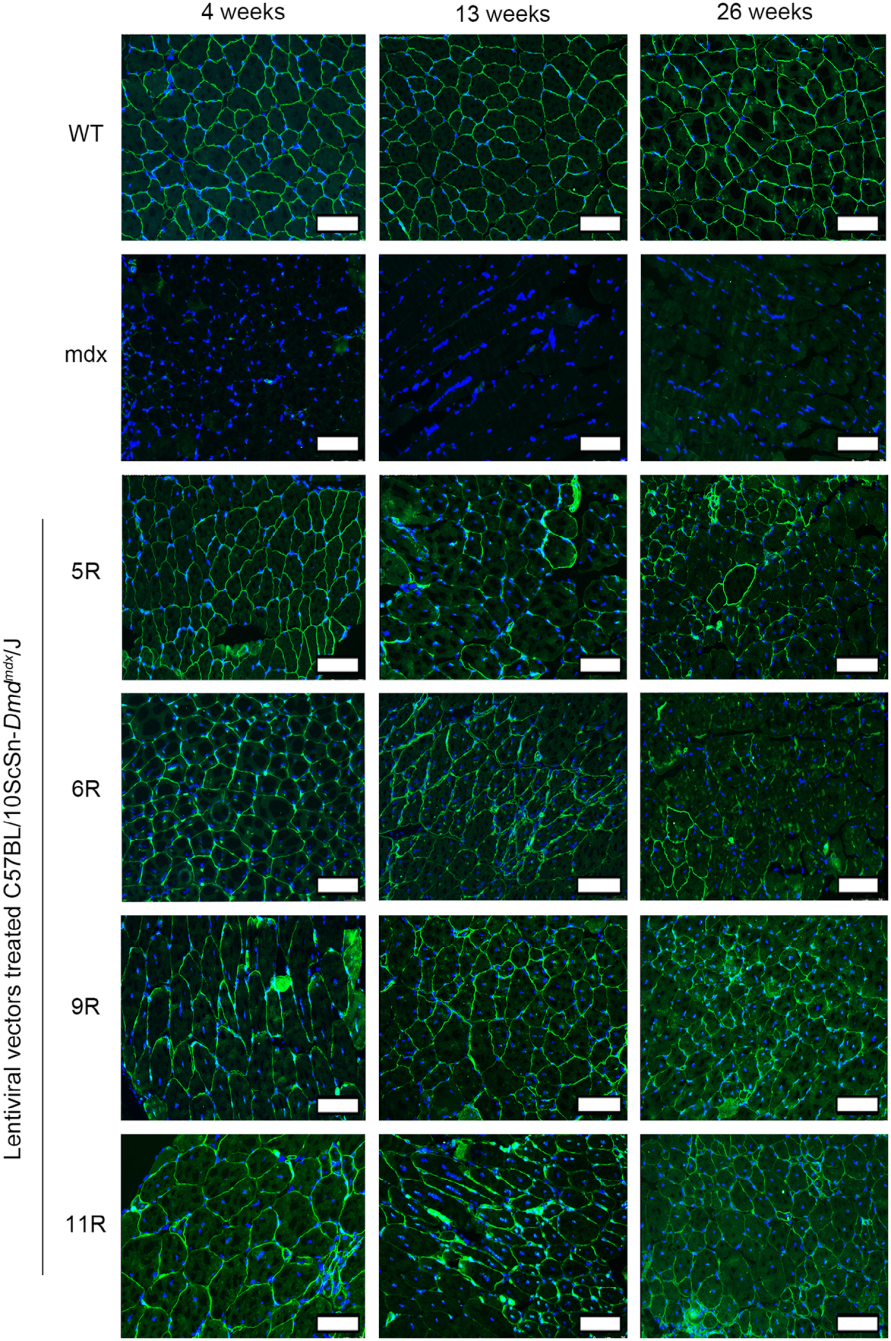
Lentiviral vectors containing modified dystrophin genes achieved the restoration of dystrophin in vivo at 4, 13, and 26 weeks after administration. Immunofluorescence staining of modified dystrophin (green) in muscle sections obtained from mice at 4, 13, and 26 weeks after treatment. Nuclei were stained with DAPI (blue). Scale bar = 100 μm. Wild type (WT): C57BL/6JNifdc mice. mdx: C57BL/10ScSn‐*Dmd^mdx^* mice. 5R, 6R, 9R, and 11R: *Lv‐Spc‐5R*‐, *Lv‐Spc‐6R*‐, *Lv‐Spc‐9R*‐, and *Lv‐Spc‐11R*‐treated groups, respectively.

We intended to explore the therapeutic effects and feasibility of LVs in DMD. In this section, the modified dystrophin expression and distribution were detected by immunofluorescence at 4, 13, and 26 weeks after treatment. The immunofluorescence images are presented in Figure 3. As could be observed in the wild‐type (WT) group, the dystrophin (green) expression in muscle tissue sections was rich and distributed on the cell membrane. In the mdx group, the case was the opposite. Consistent with a previous study,^36^ the modified dystrophin of the LV‐treated groups measurably increased and was distributed on the cell membrane, which was the same as that of the WT group. The modified dystrophin could also be stably expressed for up to 26 weeks, which might reduce the frequency of administration. And the percentage of dystrophin‐positive fibers was restored after treatment (Figure S1). However, the expression of modified dystrophin gradually decreased. This might be attributed to the immunogenicity of modified protein, and future work should focus on decreasing the immune response. In conclusion, these immunofluorescence data indicated that the LVs could deliver relatively large modified dystrophin coding sequences into muscle tissues in vivo and express protein products for at least 26 weeks.

### 2.4 Reduction in pathology following treatment

**FIGURE 4:**
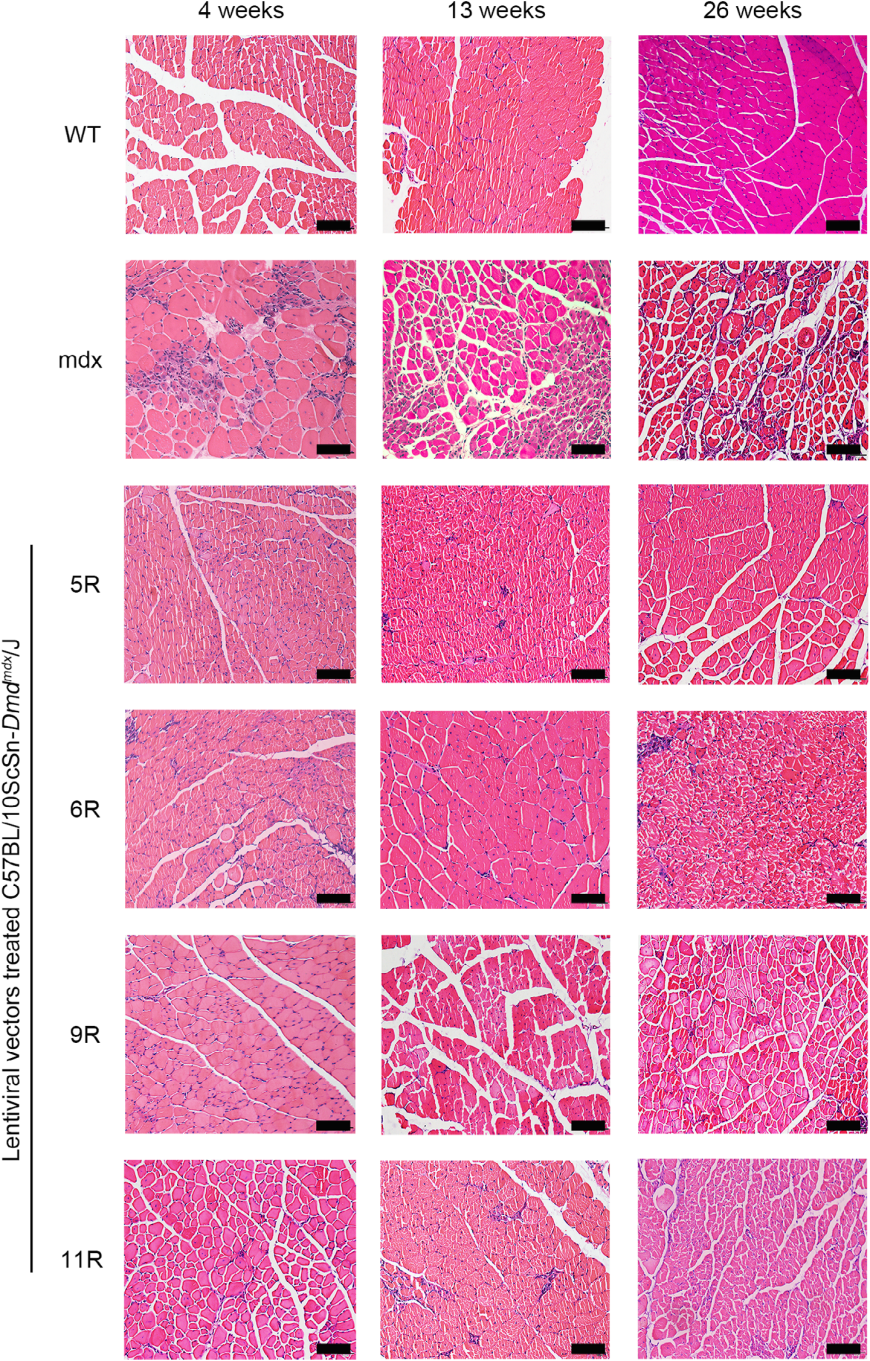
Lentiviral vectors containing modified dystrophin genes ameliorated muscle pathology in vivo at 4, 13, and 26 weeks after administration. Hematoxylin and eosin staining of tibialis anterior (TA) sections indicated the pathological alleviation in the treated groups at 4, 13, and 26 weeks after treatments (*LV‐Spc‐5R*, *LV‐Spc‐6R*, *LV‐Spc‐9R*, and *LV‐Spc‐11R*). Scale bar = 100 μm. Wild type (WT): C57BL/6JNifdc mice. mdx: C57BL/10ScSn‐*Dmd^mdx^* mice. 5R, 6R, 9R, and 11R: *Lv‐Spc‐5R*‐, *Lv‐Spc‐6R*‐, *Lv‐Spc‐9R*‐, and *Lv‐Spc‐11R*‐treated groups, respectively.

**FIGURE 5:**
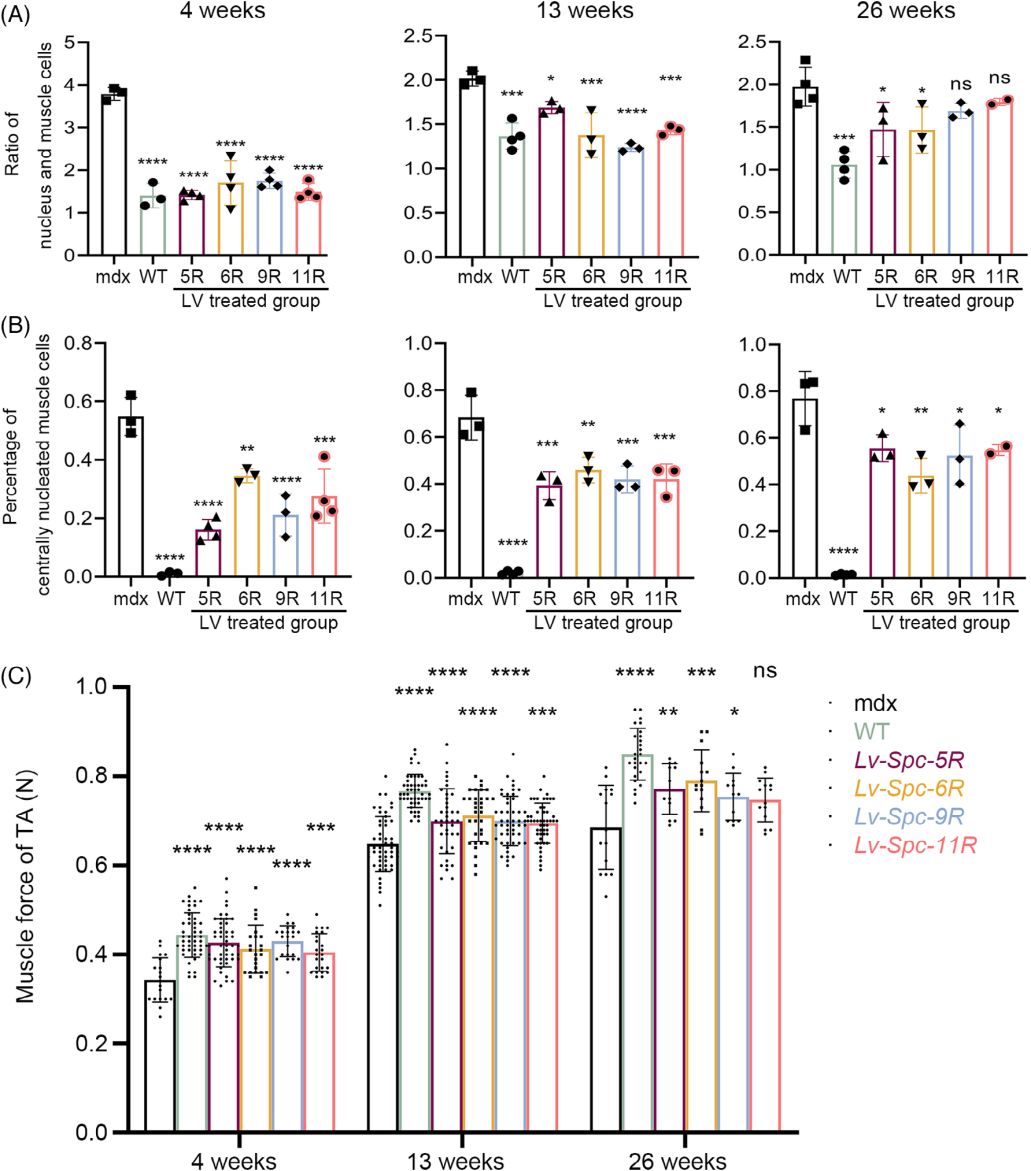
Lentiviral vectors led to the restoration of pull force in vivo. The lentiviral vector treatment ameliorated the ratio of nuclei and muscle cells (A) and the percentage of centrally nucleated muscle cells (B) in vivo. In addition, the force (C) of the treated groups was increased compared with that of the mdx group (*n* = 5−15). Data were analyzed with one‐way analysis of variance (ANOVA) and are presented as the mean ± standard deviation (SD). ns, no significance. ^*^ *p* < 0.05, ^**^ *p* < 0.01, ^***^ *p* < 0.001, ^****^ *p* < 0.0001. Wild type (WT): C57BL/6JNifdc mice. mdx: C57BL/10ScSn‐*Dmd^mdx^* mice. 5R, 6R, 9R, and 11R: *Lv‐Spc‐5R*‐, *Lv‐Spc‐6R*‐, *Lv‐Spc‐9R*‐, and *Lv‐Spc‐11R*‐treated groups, respectively.

As outlined in Section 1, we assessed the pathological alleviation of mdx mice after LV treatment. In this section, we analyzed the hematoxylin and eosin (H&E) staining images of tibialis anterior (TA) muscle tissues and measured the pull force of mice at 4, 13, and 26 weeks after treatment. The H&E images of TA and the results of the histological analysis are presented in Figures 4 and 5. The muscle tissues of the WT group presented little central nucleation and low infiltration of mononuclear cells. On the contrary, the mdx group displayed typical hallmarks of DMD muscle tissues, including a high percentage of central nucleation and high infiltration of mononuclear cells. The LV‐treated groups (including *Lv‐Spc‐5R*, *Lv‐Spc‐6R*, *Lv‐Spc‐9R*, and *Lv‐Spc‐11R*) exhibited a measurably lower ratio between nuclei and muscle cells (Figure 5A). In addition, there was a lower percentage of centrally nucleated muscle cells (Figure 5B). These results demonstrated that LVs dramatically alleviated inflammation and pathological symptoms in vivo. To further examine the reduction in pathology, the pull force was measured. As presented in Figure 5C, the pull force of the LV‐treated groups (including *Lv‐Spc‐5R*, *Lv‐Spc‐6R*, *Lv‐Spc‐9R*, and *Lv‐Spc‐11R*) was noticeably higher than that of the untreated mdx group but still lower than that of the WT group. However, the number of nuclei in treated groups gradually increased, which was always associated with the infiltration of mononuclear cells. In addition, the restoration of pull force slightly decreased. In the future, care should be taken to achieve more stable and longer therapeutic effects. It could be inferred that the LVs carrying modified dystrophin alleviated the inflammation, decreased the central nucleation cells, increased the pull force, and reduced the pathological signs in vivo.

### 2.5 A preliminary evaluation of the genetic safety of LV treatment in vivo

The property of integration into the host genome conferred the ability of long‐term transgene expression to LVs. It is also accompanied by the risk of insertion mutations, which might result in cancer. In this part, we aimed to study the genetic safety of LVs. The genomes of mice were sequenced and analyzed by Oxford Nanopore Technologies. The results consisted of insertion sites and structural variation. The insertion site information is listed in Table 1. We compared these sites with candidate proto‐oncogenes extracted from the Cancer Gene Census^37^ and did not find the vectors integrated into the candidate genes. Integrative analysis of structural variation (Figure S2) also indicated that the vectors in this study had no detectable influence on proliferation or differentiation in vivo. These results preliminarily demonstrated the genetic safety of LVs.

**TABLE 1:** Analysis of insertion sites of lentiviral vectors.

Chr	Insertion site	Regional annotation	Related transcripts	The distance from neighborhood genes	CytoBand
X	83374473	Intergenic	NONE; POU3F4	dist = NONE; dist = 133,788	Xq21.1
11	60514298	Intergenic	MS4A12; MS4A13	dist = 6870; dist = 1115	11q12.2
3	42299316	Intergenic	CCK; LYZL4	dist = 33,121; dist = 97,762	3p22.1
X	83726724	Intergenic	POU3F4; CYLC1	dist = 216,957; dist = 134,402	Xq21.1
X	83625473	Intergenic	POU3F4; CYLC1	dist = 115,706; dist = 235,653	Xq21.1
10	58877936	Intergenic	BICC1; LINC00844	dist = 46,499; dist = 121,582	10q21.1
X	85103655	Intronic	SATL1	–	Xq21.1
X	85096422	Intronic	SATL1	–	Xq21.1
14	11642200	Intergenic	NONE; NONE	dist = NONE; dist = NONE	14p11.2
X	84268742	Intergenic	MIR548I4; HDX	dist = 42,914; dist = 49,132	Xq21.1
X	83728200	Intergenic	POU3F4; CYLC1	dist = 218,433; dist = 132,926	Xq21.1
2	21766827	Intergenic	LINC01822; LINC01884	dist = 56,175; dist = 769,652	2p24.1
X	83724610	Intergenic	POU3F4; CYLC1	dist = 214,843; dist = 136,516	Xq21.1
4	59227183	Intergenic	LINC02429; MIR548AG1	dist = 180,224; dist = 1,695,436	4q13.1
X	85109413	Upstream	SATL1	dist = 445	Xq21.1
X	83135014	Intergenic	SH3BGRL; POU3F4	dist = 1,836,467; dist = 373,247	Xq21.1
X	83613929	Intergenic	POU3F4; CYLC1	dist = 104,162; dist = 247,197	Xq21.1
X	84809987	Intergenic	HDX; UBE2DNL	dist = 307,508; dist = 124,164	Xq21.1
X	84261306	Intergenic	MIR548I4; HDX	dist = 35,478; dist = 56,568	Xq21.1

*Note*: Chr—the locus of the site in chromosome.

## 3 DISCUSSION

We constructed four LVs that contained truncated and sequence‐optimized human dystrophin genes. They were injected directly into muscle tissues and stably expressed mini‐dystrophin in vivo. In addition, they exhibited the restoration of dystrophin distribution and pull force for at least 13 weeks. The mononuclear cells were reduced in the treated group. Moreover, no integration sites of vectors were distributed into oncogenes. In conclusion, this study preliminarily demonstrated the feasibility and safety of LVs with mini‐dystrophin for DMD gene therapy.

Early gene replacement or editing through AAV vectors has provided functional improvement in DMD gene therapy.^20^, ^38^, ^39^ However, the packaging ability and immunogenicity of AAV still restricted their clinical application. In contrast, the characteristics of LVs, such as large packaging capacity and low immunogenicity, raised our interest.^40^ Therefore, we administered LVs carrying a modified human dystrophin sequence to mdx mice. We found that the LVs were able to transmit the genetic material into muscle tissues, express the sequences, and alleviate the disease progression. These therapeutic effects lasted for 26 weeks in animal experiments. Apart from the restoration of muscle force, genetic safety was also appropriately assessed by sequence analysis.^41^ This study thus demonstrated the feasibility of LVs carrying mini‐dystrophin for gene replacement therapy in vivo.

LVs have been extensively used for gene transfer ex vivo. This area of research mainly focuses on genetic diseases and vaccine development. In addition, non‐integrating LVs can provide an extra level of safety by reducing the potential risks of random integration into the genome. As a result, they are considered a safer and more controllable option for clinical applications of gene editing.^42^, ^43^

Kimura et al. demonstrated that LVs carrying a μDys gene driven by the human *α‐skeletal actin* gene promoter could effectively transduce both myofibers and satellite cells.^44^ We demonstrated that truncated dystrophin genes driven by the *Spc‐5‐12* promoter could express mini‐dystrophin in vivo. In addition, our study indicated that the integration sites of vectors were not distributed into oncogenes. However, unlike in the study of Kimura et al., the therapeutic effects of our vectors only lasted for 26 weeks. This could be attributed to the *Spc‐5‐12* promoter, or the transduction of satellite cells in vivo is less efficient. The long‐term maintenance of transduced muscle satellite cells also affected the duration of benefit. Meng et al. grafted DMD myoblasts modified by LVs into an immunodeficient DMD mouse model.^36^ In contrast, we directly delivered the vectors in vivo. It has the advantages of reducing the treatment period and cost. However, this method also carries the risks of immune reactions and insufficient infection efficiency.^45^

Consistent with a previous study,^38^ we restored the dystrophin level in vivo, but the expression was low. This outcome could be attributed to the ability to infect muscle cells, the *Spc‐5‐12* promoter, or the low titer. Different from previously envisioned, the expression of mini‐dystrophin gradually decreases. This phenomenon may be attributed to the immune response triggered by the LV or the presence of the human‐derived transgene. The relatively low efficiency of infection in satellite cells or the short duration of transduced satellite cells could also play a role in the decrease.^46^, ^47^ Increasing the purity of the vectors may potentially resolve the aforementioned concerns. Further research could focus on the infection efficiency in satellite cells and the duration of transduced satellite cells in vivo.

The LVs also exhibited several advantages. One advantage of LVs was their ability to transmit larger amounts of gene material when compared with AAV vectors. Another was the LVs’ relatively low immunogenicity and the ability to carry mini‐dystrophin to reduce central nucleation in muscle tissues and improve muscle strength. These benefits represented an important step toward realizing the full potential of gene therapy for genetic diseases.

For better outcomes, further study should aim at increasing the titer and targeting of vectors. Optimization of the LV production process and higher purity were equally important. Our study provided a new option for DMD gene therapy.

## 4 MATERIALS AND METHODS

### 4.1 Mice

Mdx (C57BL/10ScSn‐*Dmd^mdx^/*J) mice were obtained from the Jackson Laboratory. In addition, C57BL/6JNifdc mice were purchased from Charles River Laboratories. Because DMD is an X‐linked recessive genetic disease, most patients were male. Hence, only male mice were used in this study. Mice were allocated at random to experimental groups. All mice were housed in specific pathogen‐free conditions at Sichuan University. Animal experiments were approved by the ethics committee of Sichuan University.

### 4.2 Cell lines and reagents

The HEK293T cell line was obtained from American Type Culture Collection. The C2C12 cell line was purchased and authenticated by the National Collection of Authenticated Cell Cultures. The cells of both lines were cultured in Dulbecco's modified Eagle medium (Gibco, #11995065) with antibiotics (HyClone, #SV30010) and 10% fetal bovine serum (FBS, Gibco, #10099141C). HEK293T cells (5 × 10^5^) were plated in a 100‐mm‐diameter cell culture dish, and the cell cultures were split (1:5) every 2 days to ensure logarithmic growth. The C2C12 cells were seeded at 5 × 10^3^ cells/cm^2^ and split (1:8) at low density to maintain an undifferentiated state.

### 4.3 Lentiviral transfer plasmids and viral production

This experiment involved the construction of four transfer plasmids, the preparation of four types of LVs, and the determination of viral vector titration. Codon optimization was performed in all of the transfer plasmids for higher expression and therapeutic effects. All the coding sequences were driven by the muscle‐ and cardiac‐specific synthetic promoter *Spc‐5‐12* to target expression. The LVs corresponding to each transfer plasmid were named *Lv‐Spc‐5R*, *Lv‐Spc‐6R*, *Lv‐Spc‐9R*, and *Lv‐Spc‐11R*. The LV envelope plasmid pMD2.G (Addgene, #12259) and the packaging plasmid psPAX2 (Addgene, #12260) were commercially available. The transfer plasmids were constructed by VectorBuilder. Self‐inactivating LVs were generated by transient co‐transfecting HEK293T cells. In brief, HEK293T cells were split to reach a confluency of 70%−90% at the time of co‐transfection and cultured in Dulbecco's modified Eagle medium (Gibco, #11995065), 1% penicillin/streptomycin (HyClone, #SV30010), and 10% FBS (Gibco, #10099141C) at 37°C and 5% CO_2_. For co‐transfection in each dish, 8 μg of pMD2.G, 8 μg of psPAX2, and 8 μg of transfer plasmids were mixed in 700 μL of Opti‐MEM, followed by the addition of 60 μg of polyethylenimine. The transfection reagents were dispersed in HEK293T cells after 20−30 min of incubation at room temperature, and the supernatant was replaced with fresh medium at 12−18 h post‐transfection. The supernatant containing LVs was collected at 48 and 72 h, followed by filtering using 0.45‐μm polyethersulfone membrane filters. Viral particles were concentrated by ultracentrifugation at 20,000 rpm for 120 min, aliquoted, and stored at −80°C. The titer of vector stocks was determined by measuring viral p24 antigen using the Lenti‐X p24 Rapid Titer Kit (Clontech, #632200).

### 4.4 In vitro viral transduction

Transduction of LVs into C2C12 myoblast cells was performed using different multiplicities of infection. C2C12 cells were seeded to reach a confluency of 70%−90% at the time of transduction and cultured in a complete medium. For transduction, concentrated LVs were dispersed in complete media with 8 μg/mL of polybrene. At 16−18 h after transduction, the supernatant was changed with a fresh complete medium, and the expression peaked at 48−72 h after transduction.

### 4.5 Western blot

Western blotting was used to determine modified dystrophin protein expression levels of viral vectors in C2C12 cells. This procedure involved protein extraction, bicinchoninic acid assay, and western blot. After transduction for 48 h, C2C12 cells were collected by scraping and centrifuged in microcentrifuge tubes. Total protein was extracted by radioimmunoprecipitation assay lysis buffer (Beyotime, #P0013B) for 15 min, and a phosphatase inhibitor cocktail (MedChemExpress, #HY‐K0010) was added at a ratio of 1:100. The extracted protein was kept on ice during handling to inhibit protein degradation. The lysis buffer–containing protein sample was centrifuged at 10,000 × *g* for 5 min to pellet the cell debris. Then, the supernatant was transferred to a clean tube and stored. The protein concentrations were determined using a bicinchoninic acid protein assay kit (Thermo Fisher Scientific, #23225). The supernatant was mixed with 5× sample loading buffer (Biosharp Life Science, #BL502B) and heated at 99°C in a metal bath for 15 min in an attempt to denature proteins. Each sample was divided into 40‐μL aliquots and stored at −80°C.

Equal amounts of protein lysates were loaded on a 7.5% sodium dodecyl sulfate–polyacrylamide gel and run at a constant voltage of 120 V for 1 h, followed by transfer onto a polyvinylidene fluoride membrane (Thermo Fisher Scientific, #88518) at 100 V for 2 h. Then, the membrane was blocked with 5% non‐fat dry milk in Tris‐buffered saline with Tween (TBST) for 3 h at room temperature. The membrane was incubated with dystrophin monoclonal antibody (Abcam, #Ab275391, 1:1000) or α‐tubulin (Cell Signaling Technology, #2144, 1:1000) overnight at 4°C. The membranes were washed with TBST three times for 6 min and incubated with a secondary antibody (Bioss, #BJ08079044, 1:8000) for 1 h at room temperature. The membranes were washed with TBST three times for 20 min and incubated with horseradish peroxidase substrate (Cofitt, #LBP7518‐50), and the proteins were detected using an eBlot Touch Imager.

### 4.6 Intramuscular injection of TA muscles

LVs were injected intramuscularly into TA muscles. Briefly, male mdx mice aged 2 days were randomly allocated into five groups (each group contained 5−15 mice) and anesthetized by hypothermia. Then, a total of 5‐μL viral preparations with a titer of 4.0–13.0 × 10^8^ IFU/mL were injected into the TA muscles of 2‐day‐old mdx mice, and phosphate‐buffered saline (PBS) was used in the mdx mice as the control group. C57BL/6JNifdc mice of the same age and sex were used as WT groups. The living conditions of the mice were monitored daily. Then, muscle tissues were harvested and recorded at 4, 13, and 26 weeks after transduction.

### 4.7 Immunofluorescence staining of muscle sections

All TA muscles were harvested from mdx or WT mice, embedded in O.C.T. compound (Sakura, #4583), and snap‐frozen. The embedded muscle samples were sectioned at a 5‐μm thickness and stored at −80°C for subsequent staining. For immunofluorescence staining, frozen sections were fixed with 4% formaldehyde (Biosharp, #BL539A) for 15 min and washed with PBS three times. The frozen sections were blocked for 1 h with 10% donkey serum (Solarbio Life Science, #SL050). Then, the sections were stained with dystrophin monoclonal antibody (Abcam, #275391, 1:200) overnight at 4°C, followed by three washes with PBS. The muscle sections were incubated with secondary antibodies (Abcam, #Ab150073, 1:100) for 1 h at room temperature, followed by three washes with PBS. The sections were then stained with 10% DAPI (4‘,6‐diamidino‐2‐phenylindole) dihydrochloride (Beyotime, #C1005) for 15 min at room temperature. Finally, the sections were observed immediately using a fluorescence microscope (Leica CM1950).

### 4.8 Hematoxylin and eosin staining

H&E staining was performed according to a routine protocol. In brief, tissues were paraffin‐embedded and sectioned. The sections were dewaxed and rehydrated, followed by staining with hematoxylin solution for 5 min. The sections were differentiated with 1% acid ethanol (1% HCl in 75% ethanol) for several seconds and rinsed in water. Then, the sections were incubated in eosin solution for several seconds and rinsed in water. Finally, the tissue sections were dehydrated in a graded series of alcohol, embedded in resin according to standard procedures, and observed.

### 4.9 Grip strength

Muscle strength was determined by a grip strength meter (Columbus Instruments, #180199). In brief, the tail of the mice was lifted at the same height as the grip strength meter grid. Then, the mice were moved horizontally and pulled away from the grid until the grasp was released. The grip strength meter automatically recorded the values of the force.

### 4.10 Statistical analysis

GraphPad Prism 8.4.0 software (GraphPad) was used for all data analyses. The D'Agostino–Pearson normality test was used to assess the normality of the data (*n* > 8). The distribution followed the normal distribution and passed the D'Agostino–Pearson normality test (*α* = 0.05). All values are expressed as the mean ± standard deviation. Significance was determined using an unpaired *t* test and one‐way analysis of variance. A value of *p* < 0.05 was considered statistically significant. In addition, the researchers were blinded to the treatment of each sample at the time assays were performed.

## AUTHOR CONTRIBUTIONS

*Conceptualization*: Z.H. *Methodology*: X.W., Y.Z., and T.L. *Software*: X.W. *Validation*: Z.H. and X.W. *Formal analysis*: X.W. *Investigation*: X.W., Ya.Z., T.L., X.D., L.Z., X.Y., and Z.H. *Resources*: Z.H. and Y.Y. *Data curation*: Z.H. and X.W. *Writing—original draft preparation*: X.W., Ya.Z., T.L., Y.F., J.Z., Y.L., Ye.Z., and Z.H. *Writing—review and editing*: Z.H. and Y.Y. *Visualization*: Z.H. *Project administration*: Z.H. and Y.Y. *Funding acquisition*: Z.H. All authors have read and agreed to the published version of the manuscript.

## CONFLICT OF INTEREST STATEMENT

All authors declare they have no conflicts of interest.

## ETHICS STATEMENT

All animal experiments were approved by the Institutional Animal Care and Use Committee of West China Hospital of Sichuan University (no. 20211343A) and performed in accordance with the guidelines of the Institutional Animal Care and Use Committee of West China Hospital of Sichuan University (Chengdu, Sichuan, China).

## Supporting information

Supporting information

## Data Availability

The data that support the findings of this study are available from the corresponding author upon reasonable request.
