# Supplementary material for: Duchenne muscular dystrophy treatment with lentiviral vector containing mini‐dystrophin gene in vivo
Source: MedComm (2020). 2024 Jan 6;5(1):e423. doi: 10.1002/mco2.423 (PMC10771042; doi:10.1002/mco2.423)
Supplement: Supplementary file 1 — Supporting information [file MCO2-5-e423-s001.docx]

**Supplemental Information for:**

**Title:** Duchenne muscular dystrophy treatment with lentiviral vector containing mini-dystrophin gene *in vivo*

Xiaoyu Wang^a^, Yanghui Zhu^a^, Taiqing Liu^a^, Lingyan Zhou^a^, Xiaodong Yang^a^, Xiangjie Di^b^, Yang Yang^a*^, Zhiyao He^a,c*^

^a^ Department of Pharmacy, Cancer Center and State Key Laboratory of Biotherapy, West China Hospital, Sichuan University, Chengdu 610041, China

^b^ Clinical Trial Center/ NMPA Key Laboratory for Clinical Research and Evaluation of Innovative Drug, West China Hospital, Sichuan University, Chengdu 610041, China

^c^ Key Laboratory of Drug-Targeting and Drug Delivery System of the Education Ministry, Sichuan Engineering Laboratory for Plant-Sourced Drug and Sichuan Research Center for Drug Precision Industrial Technology, West China School of Pharmacy, Sichuan University, Chengdu 610041, China

^*^ Corresponding author

**Correspondence to:** Yang Yang, Ph.D., professor

Zhi-Yao He, Ph.D., associate professor

Department of Pharmacy, West China Hospital, Sichuan University

No. 37 Guo Xue Xiang, Chengdu, Sichuan 610041, China

E-mail addresses: yang2012@scu.edu.cn; bachtop@163.com (Yang Yang)

zhiyaohe@scu.edu.cn; heyaode@163.com (Zhiyao He)

**Short title:** Lentiviral gene therapy for DMD


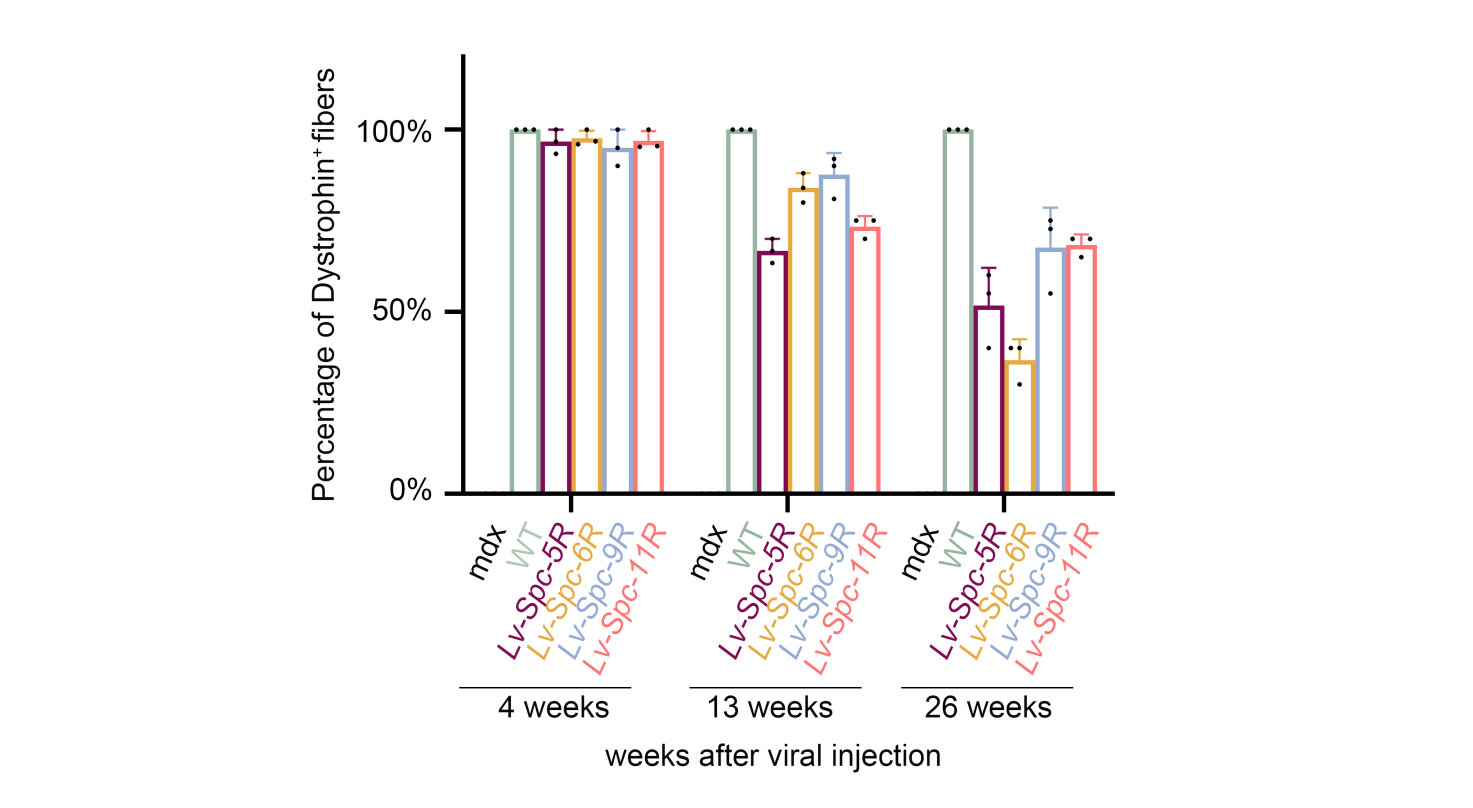


Supplemental Figure 1. The percentage of modified dystrophin expressing fibers in each treated muscle harvested at various time points postinjection. mdx: C57BL/10ScSn-*Dmd^mdx^* mice. WT (wild type): C57BL/6JNifdc mice. *LV-spc-5R, 6R, 9R*, and *11R*: *Lv-Spc-5R-, Lv-Spc-6R-, Lv-Spc-9R-, and Lv-Spc-11R*-treated groups, respectively.


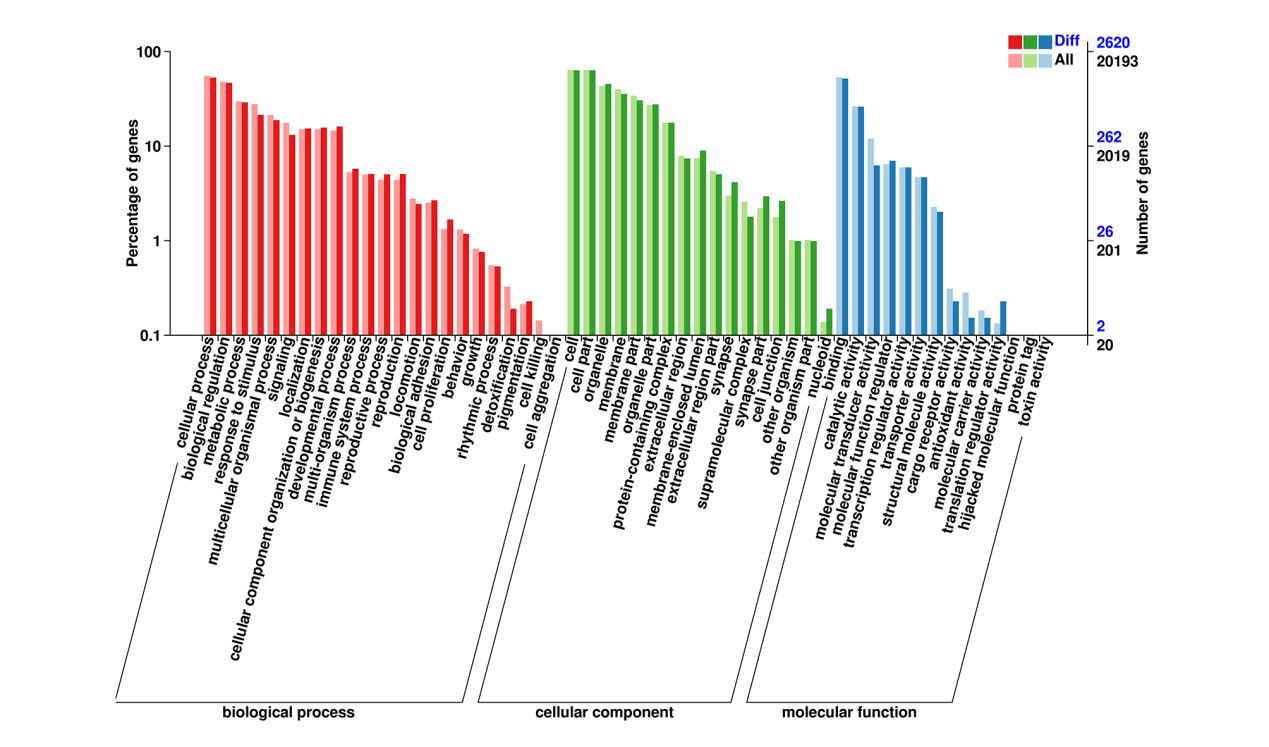


Supplemental Figure 2. The abscissa is the GO (Gene Ontology) classification, the left of the ordinate indicates the percentage of gene number, and the right of the ordinate is the number of genes. This figure presents the gene enrichment of each secondary function of GO in the background of SV (Structure Variantions)-associated genes and all genes, reflecting the status of each secondary function in the two backgrounds. The secondary function with obvious proportion difference indicates that the enrichment trend of SV-associated genes is different from that of all genes.
